# Giant hepatic hemangioma: a challenging diagnosis

**DOI:** 10.11604/pamj.2021.38.354.25620

**Published:** 2021-04-13

**Authors:** Sana Landolsi, Imène Ridène

**Affiliations:** 1 Department of Surgery, Mahmoud El Matri Hospital, Ariana, Faculty of Medicine, University of Tunis El Manar, Tunis, Tunisia,; 2 Department of Radiology, Mahmoud El Matri Hospital, Ariana, Faculty of Medicine, University of Tunis El Manar, Tunis, Tunisia

**Keywords:** Giant hemangioma, cavernous liver hemangiomas, treatment

## Image in medicine

A 50-year-old man without medical history, complained about effort dyspnea with enlargement of the abdomen. Physical examination showed an enlarged liver reaching the right and the left iliac fossa with a collateral venous circulation. Biological exams were within normal ranges. Hepatitis B and C serologies were negative. Abdominal computed tomography and magnetic resonance imaging demonstrated an enlarged liver of 36 cm in diameter. Multiple heterogenous bilateral liver masses with enhancement after contrast intravenous injection were shown.The largest one with 26 cm in diameter was located in the left liver. A dense collateral venous circulation existed. A diagnostic percutaneous biopsy was made. Histopathological exam concluded to giant cavernous cavernoma. Since the surgery was risky especially hemorrhage, a percutaneous embolization for the largest mass was indicated.

**Figure 1:**
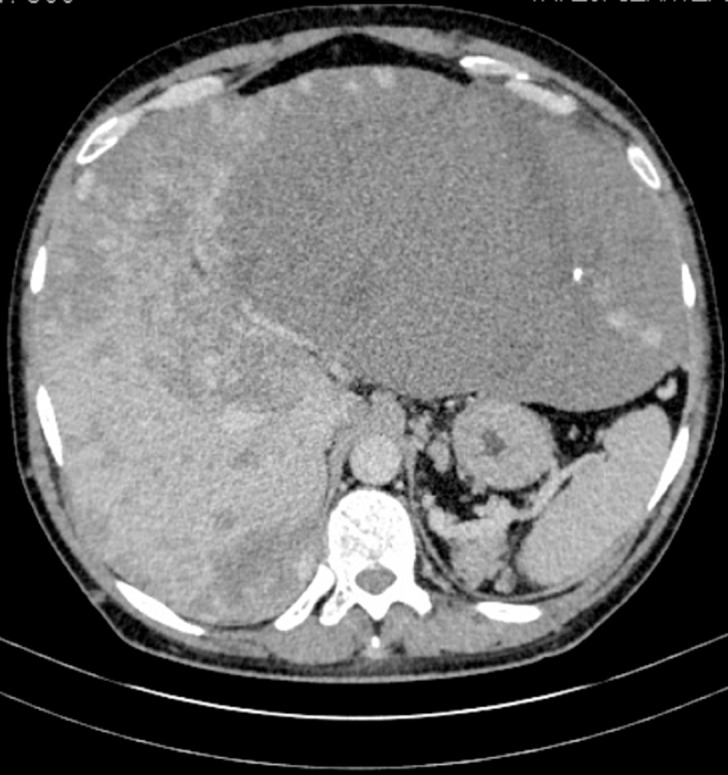
giant cavernous hemangioma

